# The anti-diabetic and antioxidant effects of a combination of *Commiphora mukul*, *Commiphora myrrha* and *Terminalia chebula* in diabetic rats

**Published:** 2019

**Authors:** Reyhaneh Sotoudeh, Mousa-Al-Reza Hadjzadeh, Zahra Gholamnezhad, Azita Aghaei

**Affiliations:** 1 *Division of Neurocognitive Sciences, Psychiatry and Behavioral Sciences Research Center, Mashhad University of Medical Sciences, Mashhad, Iran.*; 2 *Department of Physiology, Faculty of Medicine, Mashhad University of Medical Sciences, Mashhad, Iran.*; 3 *Neurogenic Inflammation Research Center, Mashhad University of Medical Sciences, Mashhad, Iran.*; 4 *Pharmacological Research Center of Medicinal Plants, Mashhad University of Medical Sciences, Mashhad, Iran.*

**Keywords:** Anti-diabetic, Hypolipidemic, Antioxidant, Commiphora mukul, Commiphora myrrha, Terminalia chebula

## Abstract

**Objective:**

Effects of *Commiphora mukul* and *Commiphora myrrha* ethanolic extracts and *Terminalia chebula* hydro-ethanolic extract combination were evaluated in streptozotocin (STZ)-induced diabetic rats.

**Materials and Methods:**

Male Wistar rats (n=48) were randomly assigned into: control; diabetic; diabetic+metformin (300 mg/kg); diabetic+dose 1 of herbal combination (438 mg/kg of *C. mukul*+214 mg/kg of *C. myrrha*+857 mg/kg of *T. chebula*); diabetic+dose 2 (642 mg/kg of *C. mukul*+214 mg/kg of *C. myrrha*+642 mg/kg of *T. chebula*); and diabetic+dose 3 (857 mg/kg of *C. mukul*+438 mg/kg of *C. myrrha*+1714 mg/kg t of *T. chebula*). All treatments were given orally by gavage. Diabetes was induced by STZ (60 mg/kg, i.p.). At the end of study (day 28), blood glucose, insulin and lipid profile; as well as hepatic malondialdehyde (MDA) and thiol content, and superoxide dismutase (SOD) and catalase (CAT) activities were determined.

**Results:**

In diabetic rats, plasma glucose, triglycerides (TG), total cholesterol (TC), and LDL-C, as well as hepatic MDA levels were elevated but plasma HDL-C and insulin, and hepatic thiol content and SOD and CAT activities were reduced compared to control (p<0.01-p<0.001). In diabetic+dose 3, plasma TC, TG, and LDL-C and hepatic MDA level decreased (p<0.001), while plasma HDL-C and insulin, and hepatic thiol content, and SOD and CAT activities increased compared to diabetic (p<0.01-p<0.001). Treatment with dose 1 and 2 improved such abnormalities in diabetic rats except for insulin level (p<0.05-p<0.001). The herbal combination effects were comparable to those of metformin. Metformin did not significantly change serum insulin and HDL-C levels, and hepatic SOD activity; however, serum levels of TC, TG, and LDL-C, as well as hepatic MDA levels, thiol content and CAT activity were improved compared to diabetic (p<0.05-p<0.001).

**Conclusion:**

These results indicate that this herbal combination acts as an anti-diabetic, antioxidant and hypolipidemic agent and it may be suggested as a beneficial remedy for diabetic patients.

## Introduction

Diabetes is known as one of the world’s most common endocrine disorder and has been a major non-communicable disease over recent decades. The worldwide incidence of diabetes is 451 million which is likely to reach to 693 million in 2045 (Cho et al., 2018 ▶). Diabetes mellitus as a group of metabolic diseases, is mainly manifested by elevated plasma glucose level resulting from defects in insulin secretion and/or action or both (Bhat et al., 2011 ▶). Exposure to chronic hyperglycemia can cause macro and micro vascular damages. Cardiovascular disease like myocardial infarction and stroke during diabetes are the major macro vascular complications, long-term hyperglycemia also increases ROS (Reactive Oxygen Species) production (King and Loeken, 2004 ▶). Elevation of oxidative stress and changes in antioxidant enzyme activity might play a main role in the development of micro vascular complications (Moussa, 2008 ▶). Diabetic retinopathy, nephropathy and neuropathy are main micro vascular complications of untreated diabetes (Ighodaro and Adeosun, 2018 ▶). Patients with diabetes mellitus frequently exhibit central obesity, dyslipidemia, insulin resistance and hypertension (Jandeleit-Dahm and Cooper, 2002 ▶; Pinhas-Hamiel and Zeitler, 2007 ▶). Several types of anti-diabetic drugs have been produced and are in use for diabetic patients. Many of anti-diabetic synthetic drugs are too expensive (especially for people living in developing countries) or have undesirable side effects. Therefore, several medicinal plants with high anti-hyperglycemic activity are used for treatment of diabetes. These herbal remedies may slow down the development of complications in diabetic patients and are regarded as low-cost treatments with minimal or no side effects (Shukla et al., 2000 ▶).

Metformin is one of the first-line drugs for type 2 diabetes treatment (Zhou et al., 2018 ▶). Metformin ameliorates hyperglycemia by decreasing production of hepatic glucose (Hundal et al., 2000 ▶) and increasing glucose utilization by skeletal myocytes (Turban et al., 2012 ▶). Therefore, in animal investigations, anti-diabetic effects of given agents/compounds can be compared to those of metformin as a standard drug.

Several plants extracts with anti-diabetic properties have been proposed. Plant derived drugs are frequently considered to be cheaper and less toxic, and have fewer side effects compared to synthetic ones (Nasri, 2013 ▶). *Commiphora myrrha* (*C. myrrha*) is native to Northeastern Africa (Helal et al., 2005 ▶). Its aromatic gum resin has been widely used for treatment of rheumatoid arthritis (Su et al., 2015 ▶), sinusitis and cough, gum and gingival problems, sore throat, gastrointestinal tract disorders and diarrhea, asthma and traumatic injuries (El Ashry et al., 2003 ▶; Shen; et al., 2012 ▶). *C. myrrha* also exhibits anti-diabetic (Helal; et al., 2005 ▶), hypolipidemic (Shen; et al., 2012 ▶; Shalaby and Hammouda, 2014 ▶) and antioxidant (Racine and Auffray, 2005 ▶) activities. In alloxan-induced diabetic rats, the aqueous extract of *C. myrrha* reduced hyperglycemia by increasing the serum level of insulin (Helal et al., 2005 ▶). It also reduced animal's weight gain and improved lipids profile and hyperlipidemia in obese rats (Shalaby and Hammouda, 2014 ▶).

Another anti-diabetic plant is* Commiphora mukul* (*C. mukul*) growing in the dry regions of India; a gum resin called “*guggul*” is obtained from *C. mukul* bark and has anti-diabetic properties (Ramesh et al., 2012 ▶). The gum resin extract of *C. mukul *possesses anti-inflammatory (Francis et al., 2004 ▶), antispasmodic, carminative, antiseptic, sedative, diaphoretic, diuretic, expectorant, aperient, thyroid stimulant, demulcent, aphrodisiac (Shen et al., 2012 ▶), hypolipidemic, antioxidant (Singh et al., 1994 ▶; Sharma et al., 2009 ▶), hypoglycemic and anti-diabetic (Bellamkonda et al., 2011 ▶) activities. Two stereoisomers, Z- and E-guggulsterone (trans- and cis-4,17 (20)-pregnadiene-3, 16-dione, respectively), from *C.* *mukul* were indicated to be responsible for hypolipidemic activity mediated via the farnesoid X receptor (FXR) antagonizing, which lead to preventing inhibitory feedback of bile acid synthesis (Niethammer et al., 2009 ▶). It showed protective effects against oxidative damage in streptozotocin (STZ)-induced diabetic rats (Ramesh et al., 2012 ▶; Ramesh and Saralakumari, 2012 ▶). This activity may contribute to the plant’s effects in terms of lowering lipid peroxidation and enhancing the antioxidant defense (Ramesh et al., 2012 ▶).

*Terminalia chebula *(*T. chebula*) is a plant with anti-diabetic and hepatoprotective effects. The plant is native to Southeast Asia and India, and has been reported to exhibit several pharmacological properties including anti-bacterial (Kannan et al., 2009 ▶), anti-allergic, antifungal, wound healing, anti-cancer, anti HIV, anti-mutagenic (Lee et al., 2005 ▶), antioxidant (Naik et al., 2004 ▶), anti-diabetic (Kumar et al., 2006 ▶) and maltase inhibitory activity (Gao et al., 2007 ▶). Several chemical compounds including tannins, polyphenols and triterpenoids, were isolated from *T. chebula* (Pfundstein et al., 2010 ▶). These compounds are powerful antioxidant and anti-inflammatory agents (Sheng et al., 2018 ▶). Several studies showed the antioxidant effects of *T. chebula* (Naik et al., 2004 ▶; Lee et al., 2007 ▶). In this regard, *T. chebula* extract was showed to have free radical scavenging activity and inhibition of lipid peroxidation in a hepatic injury model. The hepatoprotective effect of *T. chebula* in liver injury was attributed to its antioxidant capacities and modulation of inflammatory reactions (Naik et al., 2004 ▶; Choi et al., 2015 ▶).

In traditional medicine, *C. mukul, C. myrrha*, and *T. chebula* were extensively used as anti-diabetic remedy due to their antioxidant, hypoglycemic, and hypolipidemic properties. In traditional Persian medicine, a combination of these plants was recommended for the diabetic patients (Shokoohi et al., 2017 ▶). Therefore, the aim of this study was to investigate the effects of a combination of ethanolic extracts of *C. mukul* and *C. myrrha* and hydro-ethanolic extract of *T. chebula* on serum glucose and insulin levels, serum lipid profile, as well as lipid peroxidation, and antioxidant enzymes activities in the liver, and body weight changes in STZ-induced diabetic rats.

## Materials and Methods

**Preparation of extracts**

Here, 50 g of the plant (gum resin of *C. mukul*), voucher specimen No. E1025-FUMH, was washed with drinking water, dried in dark place at room temperature, and powdered. Ethanolic extract was prepared by soaking gum resin powder in ethanol (1500 ml) with daily shaking for 7 days. The extract was filtered and concentrated using a rotatory evaporator (Ramesh, Karuna et al., 2013 ▶). Herb-to-product ratio was 5:3. Similarly, 50 g of *C. myrrha*, voucher specimen No. E1026-FUMH, resin was macerated in ethanol (1500 ml) with daily shaking for 3 days. Then, the extract was filtered, concentrated under reduced pressure and finally dried in vacuum desiccators (Shalaby and Hammouda, 2014 ▶). Herb-to-product ratio was 5:2. The fruits of *T. chebula* (100 g), voucher specimen No. E1024-FUMH, were powdered and macerated in 1800 ml ethanol:water (70:30, v/v) for 72 hours. Then, the extract was filtered and concentrated using a rotatory evaporator (Ahmadi-Naji et al., 2017 ▶). Herb-to-product ratio was 10:7. Finally, the obtained extracts were stored at 4°C for further use.

**Animals**

A total of 48 male Wistar rats (250±25 g body weight) were applied in this study. The animals were purchased from the Animal House of Mashhad University, and kept under standard conditions (at 22±2°C temperature with 12hr- 12hr light-dark cycles) and had free access to food and water, during the experiment. The study was carried out in accordance with ethical principles and policies approved by the Committee on Animal Research of Mashhad University of Medical Sciences (Ethical No. 951850).

**Induction of diabetes**

In overnight fasted rats, diabetes was induced through a single intraperitoneal injection of STZ (60 mg/kg) solution which was prepared freshly. The rat's plasma glucose levels were determined 72 hr after STZ injection to confirm diabetes induction. Fasting plasma glucose level more than 250 mg/dl in rats were considered as diabetes marker and those animals were used in the experiment.

**Experimental design**

Animals were randomly divided into: non-diabetic control (C); diabetic animals (D); metformin (300 mg/kg) treated diabetic animals (D+M); diabetic animals treated with 438 mg/kg ethanolic extract of *C. mukul*+214 mg/kg ethanolic extract of *C. myrrha*+857 mg/kg hydro-ethanolic extract of *T. chebula* (D+dose 1); diabetic animals treated with 642 mg/kg ethanolic extract of *C. mukul*+214 mg/kg ethanolic extract of *C. myrrha*+642 mg/kg hydro-ethanolic extract of *T. chebula* (D+dose 2); and diabetic animals treated with 857 mg/kg ethanolic extract of *C. mukul*+438 mg/kg ethanolic extract of *C. myrrha*+1714 mg/kg hydro-ethanolic extract of *T. chebula *(D+dose 3). Groups C and D were treated with saline, and all treatments were given orally by gavage for 4 weeks. The body weight was measured at the beginning and the end of the experiment. At the end of the experiment, rats were fasted overnight and sacrificed under ether anesthesia. Blood was collected from retro-orbital plexus of the animals. The abdomen was cut open and liver tissue was collected.

**Chemicals**

Kits used for determination of glucose, triglycerides (TG), total cholesterol (TC), low-density lipoprotein cholesterol (LDL-C), high density lipoprotein cholesterol (HDL-C) levels, were purchased from Pars Azmoon Co. (Iran, Tehran). Plasma insulin levels were measured using enzyme immunoassay kits according to the manufacturer’s instructions (Cayman Chemical, USA). STZ were obtained from Sigma Chemical Co. (St. Louis, MO, USA). Disodium salt of ethylene diamine tetra-acetic acid (EDTA), 5, 5-dithiobis-(2-nitrobenzoic acid) (DTNB), and 1-chloro-2, 4-dinitrobenzene (CDNB) were purchased from Merck Company.

**Measurement of oxidative stress markers**

MDA level was measured by Kaveh et al. 2017 method based on MDA reaction with thiobarbituric acid (TBA), which produces a pink complex with a peak absorbance at 535 nm (Kaveh et al., 2017 ▶). Total thiol content was measured by the method of Ellman. SH groups produce a yellow complex which has a peak absorbance at 412 nm. (Habeeb, 1972 ▶). The SOD activity was determined by the method of Madesh and Balasubramanian. The procedure involving production of superoxide through auto-oxidation of pyrogallol and the inhibition of superoxide-dependent reduction of the tetrazolium dye, MTT (3-(4, 5-dimethylthiazol-2-yl) 2, 5-diphenyltetrazolium bromide) conversion to formazan (Madesh and Balasubramanian, 1997 ▶). The activity of CAT was determined according to the method of Aebi. Hydrogen peroxide (H2O2) was used as a substrate. The reaction was started by adding H2O2 and reduction of absorption was measured at 240 nm (Aebi, 1984 ▶).

**Statistical analyses**

Data were showed as mean ± SEM. One-way analysis of variance (ANOVA) followed by Tukey's *post-hoc* test was used for statistical analysis. P values <0.05 were considered significant.

## Results

**Effect of metformin and herbal treatment on body weight changes**

**Figure 1:**
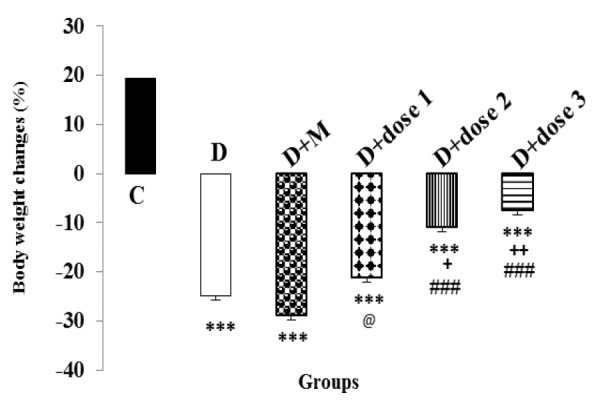
Effect of metformin and herbal treatment on body weight changes (%) in control (C), diabetic (D), diabetic+metformin (D+M), and diabetic+3 dose of herbal combination (doses 1, 2 and 3) groups

At the end of the study period (day 28), the body weight of diabetic animals and groups treated with herbal combination (doses 1, 2 and 3) and metformin showed significant (all, p<0.001) reductions compared to C group. Significant (p<0.05-p<0.01) increases in body weight were found in D+dose 2 and D+dose 3 groups compared to D-group. There were non-significant changes in body weight in D+M and D+dose 1 groups compared to D-group. The body weights of D+dose 2 and D+dose 3 groups showed significant (p<0.001) increases in compared to D+M group. Significant higher increases (p<0.05) in body weight was found in D+dose 3 group compared to D+dose 1 group (Figure 1). 

**Effect of metformin and herbal treatment on fasting blood glucose level**

**Figure 2:**
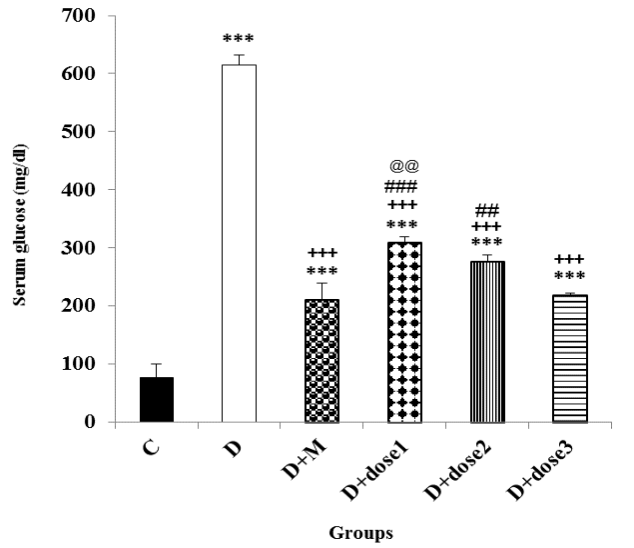
Effect of metformin and herbal treatment on plasma glucose levels in control (C), diabetic (D), diabetic+metformin (D+M), and diabetic+3 dose of herbal combination (doses 1, 2 and 3) groups

Four weeks after induction of diabetes, measurement of blood glucose levels showed significant hyperglycemia in diabetic animals compared to control group (p<0.001). At the end of the experiment, serum glucose levels in groups treated with herbal combination (doses 1, 2 and 3) and metformin were significantly (p<0.001 for all cases) lower than that of D-group. The reduction in serum glucose levels in D+dose 3 group was comparable to that of the D+M group. However, serum glucose levels in groups treated with herbal combination (doses 1, 2 and 3) and metformin were still significantly (all, p<0.001) higher than control group. Serum glucose level in D+dose 3 group was significantly (p<0.01) lower than D+dose 1 group (Figure 2).

**Effect of metformin and herbal treatment on serum insulin level**

**Figure 3:**
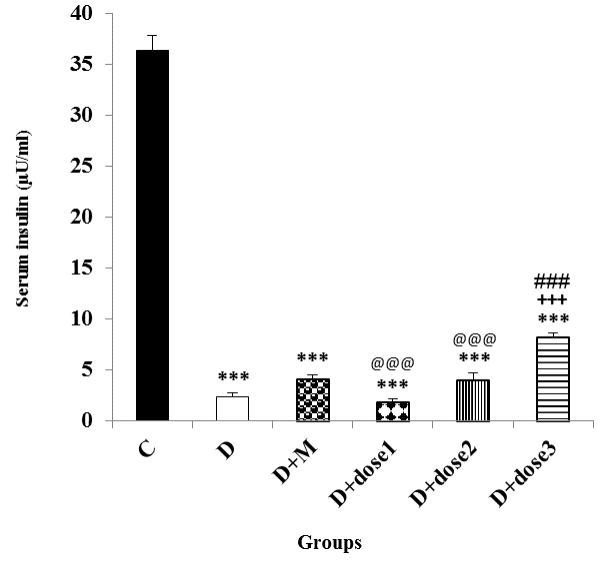
Effect of metformin and herbal treatment on plasma insulin levels in control (C), diabetic (D), diabetic+metformin (D+M), and diabetic+3 dose of herbal combination (doses 1, 2 and 3) groups

As shown in Figure 3, D-group and all groups treated with herbal combination exhibited a marked (p<0.001) decrease in serum insulin levels compared to control group. At the end of the experimental period, the serum level of insulin in D+dose 3 group was significantly (p<0.001) higher than that of the D-group but still significantly (p<0.001) lower than the control group. There was an insignificant change in serum level of insulin in the D+dose 1 and D+dose 2 groups compared to D-group. The serum insulin level in the D+dose 3 group showed a significant (p<0.001) increase in comparison to D+M, D+dose 1 and D+dose 2 groups.

Values are presented as mean±SEM. Groups D, D+M, D+dose 1, D+dose 2 and D+dose 3 *vs* group C: ^***^p<0.001. Groups D+dose 2 and D+dose 3 *vs* group D: ^+^p<0.05; ^++^p<0.01. Groups D+dose 2; D+dose 3 *vs* group D+M: ^###^p<0.001. Group D+dose 1 *vs* group D+dose 3: ^@^p<0.05.

Values are expressed as mean±SEM. Groups D; D+M; D+dose 1; D+dose 2; D+dose 3 *vs* group C: ^***^p<0.001. Groups D+M; D+dose 1; D+dose 2; D+dose 3 *vs* group D: ^+++^p<0.001. Groups D+dose 1; D+dose 2 *vs* group D+M: ^###^p<0.001. Group D+dose 1 *vs* group D+dose 3: ^@@^p<0.01.

Values are presented as mean±SEM. Groups D, D+M, D+dose 1, D+dose 2 and D+dose 3 *vs* group C: ^***^p<0.001. Group D+dose 3 *vs* group D: ^+++^p<0.001. Group D+dose 3 *vs* group D+M: ^###^p<0.001. Groups D+dose 1 and D+dose 2 *vs* group D+dose 3: ^@@@^p<0.001.

**Effect of metformin and herbal treatment on serum lipid profile**

In diabetic animals, there was a significant increase in serum TC, TG, and LDL-C (all, p<0.001) but a significant decrease (all, p<0.001) in HDL cholesterol (HDL-C) were observed compared to control group. Serum TG, TC, and LDL-C levels were decreased significantly (all, p<0.001) in D+M and D+dose 1, 2 and 3 groups as compared to D group. Moreover, serum level of HDL-C significantly increased (both, p<0.001) in D+dose 2 and D+dose 3 when compared to D group. Serum HDL-C level was not significantly changed in D+M and D+dose 1 compared to D group. Serum levels of TG, TC, and LDL-C showed insignificant changes in D+dose 2 and D+dose 3 groups as compared to control group. Serum TG and LDL-C levels were reduced significantly (p<0.001-p<0.05) in D+dose 3 as compared to D+M group. There was a significant increase (both, p<0.001) in serum level of HDL-C in D+dose 2 and D+dose 3 groups compared to the D+M group. The effect of D+dose 3 on serum TG was significantly higher (p<0.001) than D+dose 1 and D+dose 2 (Table 1).

**Table 1:** Lipid profile in control (C), diabetic (D), diabetic+metformin (D+M), and diabetic+3 dose of herbal combination (doses 1, 2 and 3) groups

Variable (mg/dl)	Group C	Group D	Group D+M	Group D+dose 1	Group D+dose 2	Group D+dose 3
Total Cholesterol	85.31±0.83	102.27±2.31***	73.39±1.46+++	77.07±0.4*,+++	84.56±1.97+++	83.71±3.83+++
HDL- Cholesterol	58.14±1.27	42.5±2.35***	45.83±0.89*	52±2.42	63.28±4.14+++,###	66±2.22+++,###
LDL- Cholesterol	17.57±0.49	23.75±1.14***	16.83±0.87+++	13.5±0.77+++	13.28±1.14+++,#	12.25±0.79+++,##
TG	48.02±1.53	180.12±9.31***	53.67±3.07+++	57.86±2.1+++,@@@	40.01±1.17+++,@@@	27.33±1.14+++,###

Values are presented as mean ±SEM. Groups D, D+M, D +dose 1, D +dose 2 and D +dose 3 *vs* group C:

Groups D+M, D +dose 1, D +dose 2 and D +dose 3 *vs* group D:

Groups D +dose 2 and D +dose 3 *vs* group D+M:

Groups D+dose 1 and D +dose 2* vs* group D+dose 3:

* p<0.05;

** p<0.01;

*** p<0.001.

+++ p<0.001.

# p<0.05;

## p<0.01.

@@@ p<0.001.

**Effect of herbal treatment and metformin on liver antioxidants CAT and SOD and levels of MDA and content of total thiol**

Table 2 summarizes MDA levels, total thiol content and antioxidant activities of SOD and CAT in the liver of different groups of the animals. A significant increase (p<0.001) in MDA levels of the liver but a significant decrease (p<0.001) in total thiol content was detected in diabetic animals compared to C group. Diabetic animals showed significant decreases in the CAT and SOD (p<0.001-p<0.01) activities as compared to the C group. Treatment of diabetic rats with herbal combination of doses 1, 2 and 3 and metformin for 4 weeks lead to a significant decrease (all, p<0.001) in liver MDA level. Total thiol content and CAT activity significantly (p<0.001-p<0.05) increased in all herbal-treated groups compared to the D group. Significant increase (p<0.01) in SOD activity were only observed in D+dose 3 compared to the D group. Total thiol content showed a significant increase (p<0.001) but MDA level exhibited a significant decrease (p<0.001) in D+dose 3 compared to the D+M group. MDA level was significantly lower (p<0.01) and CAT activity significantly higher (p<0.01) in D+dose 3 group compared to D+dose 1 group. Significant higher (p<0.001) total thiol content was found in D+dose 3 group compared to D+dose 1 and D+dose 2 groups.

**Table 2:** Liver oxidative stress markers and antioxidants in control (C), diabetic (D), diabetic+metformin (D+M), and diabetic+3 dose of herbal combination (doses 1, 2 and 3) groups

Variable	Group C	Group D	Group D+M	Group D+dose 1	Group D+dose 2	Group D+dose 3
MDA concentration (nmol/g liver)	66.87±2.31	124.12±4.46***	81.62±1.14***,+++	74.25±1.64+++,@@	70.87±1.02+++	67.00±1.14+++,###
Total thiol (µmol/g liver)	2.97±0.02	1.89±0.04***	2.58±0.07***,+++	2.49±0.05***,+++,@@@	2.57±0.03***,+++,@@@	2.89±0.03+++,###
SOD activity (U/g liver)	27.62±1.46	18.87±1.71***	22.5±1.28	23.12±1.85	25.5±1.55	27.12±1.48+++
CAT activity (U/g liver)	0.92±0.05	0.56±0.03***	0.81±0.05++	0.75±0.02+,@@	0.84±0.04+++	0.9±0.03+++

Values are presented as mean±SEM. Groups D, D+M, D+dose 1 and D+dose 2 *vs* group C:

Groups D+M, D+dose 1, D+dose 2 and D+dose 3 *vs* group D:

Group D+dose 3 *vs* group D+M:

Group D+dose 1 *vs* group D+dose 3:

Groups D+dose 1 and D+dose 2* vs* group D+dose 3:

*** p<0.001.

+ p<0.05;

++ p<0.01;

+++ p<0.001.

### p<0.001.

@@ p<0.01,

@@@ p<0.001.

## Discussion

In diabetes mellitus, the homeostatic status of carbohydrate, lipid and protein metabolism that is regulated through the insulin action, is disturbed and results in increased blood glucose levels (Sharma et al., 2009 ▶). Persistent hyperglycemia resulted in different acute and chronic complications. The number of diabetics worldwide is increasing.

STZ-induced diabetes is accompany with a marked decline in body weight (Bellamkonda et al., 2011 ▶), which was also observed in the present study. Diabetic rats treatment with dose 2 and dose 3 of herbal combination significantly prevented the body weight loss; although, it was not restored to control levels. In diabetes, increase in muscle wasting and tissue protein degradation may lead to weight loss (Swanston-Flatt et al., 1990 ▶). In Helal et al. study, the body weight of alloxan-induced diabetic rats increased significantly after treatment with *C. myrrha* extract. They argued that this may be induced by stimulation of carbohydrate metabolism (Helal et al., 2005 ▶). Alcoholic extract of *C. mukul *gum resin could prevent weight loss in diabetic rats. The authors attributed this effect to the enhancement of insulin secretion, which is the main glycogenolysis regulator in the liver and muscle tissues (Bellamkonda et al., 2011 ▶).

The results of the present study also showed that blood glucose levels significantly decreased in groups treated with herbal combination of *C. mukul C. myrrha, *and* T. chebula* and metformin but remained significantly higher than control group. The improvement of serum glucose level in D+dose 3 was similar to that of the metformin-only treated group which shows that antihyperglycemic effect of these medicinal plants is comparable to metformin. Plasma insulin level decreased significantly in diabetic rats. Hypoinsulinemia was caused by the selective, destructive and cytotoxic effect of STZ on the B-cells of rats’ pancreas and hyperglycemia was due to decrement of insulin secretion (Szkudelski, 2001). Figures 2 and 3 show that dose 3 of herbal combination has marked hypoglycemic and hyperinsulinemic activity in diabetic animals. These effects were shown in diabetic rats which were treated with* C. myrrha* extract which was attributed to phytosterols or polysaccharides content (Helal et al., 2005 ▶). Diabetic rats treated with* C. mukul *gum resin extract, another component of the herbal combination, also indicated significant decline in the plasma glucose content but significant increases in the plasma insulin level. These effects might be attributed to the pancreatic insulin secretion by the remnant B-cells or insulin release from its bound form (Bellamkonda et al., 2011 ▶). Stimulation of insulin secretion from the existing or regenerated B-cells by* T. chebula* extract caused elevation of plasma insulin levels that resulted in decreased blood glucose content (Kumar et al., 2006 ▶). *T. chebula *showed α-glucosidase inhibitory activity that was attributed to its tannins. Inhibition of intestinal α-glucosidase enzyme results in reduction of digestion and absorption of starch, subsequently lowering the postprandial hyperglycaemia related responses (Sasidharan et al., 2012 ▶). This finding is in favor of the results of the present study. The herbal combination used in our study had all these activities and it could influence the glucose and insulin levels. These results confirm our study outcomes that a mixture of *C. mukul*,* C. myrrha*, and *T. chebula *extracts can improve plasma insulin level and restore plasma glucose level to almost normal values. In addition, the hypoglycemic activity of the herbal combination was reported in a clinical trial; however, the plasma insulin level was not determined (Shokoohi et al., 2017 ▶).

Our results showed severe alterations in the serum lipid profile in diabetic animals that were previously reported to happen frequently in STZ-induced diabetic rats (Pushparaj et al., 2000 ▶, Ramesh et al., 2013 ▶). There was significant elevation in the levels of total serum cholesterol, LDL-C and triglyceride, and a significant depression in the level of HDL-C in diabetic rats compared to control group. The plasma lipid profile in was improved significantly in diabetic rats treated with herbal combination (*C. mukul+C. myrrha+T. chebula*) and metformin. The hypolipidemic effects of *C. mukul* and its well-known components (E- and Z-guggulsterone), were shown by various experiments in hyperlipidemic animals (Urizar et al., 2002 ▶; Sharma et al., 2009 ▶). Beneficial effects of *C. mukul* and its components on dyslipidemia may be due to the cholesterol synthesis inhibition in the liver by inhibition of the farnesoid X receptor (FXR) (Urizar et al., 2002 ▶). *T. chebula *extract administration substantially improved dyslipidemia in diabetic rats. STZ-induced diabetic rats treated with the ethyl acetate fraction of *T. chebula *fruit ethanolic extract, indicated hypolipidemic effect of this plant (Kim et al., 2011 ▶). Our findings have been approved by previous studies reporting hypolipidemic effects of *C. mukul, C. myrrha *and* T. chebula*.

Our findings showed elevation of lipid peroxidation and decrement of antioxidant potential in the liver of diabetic animals which might result in cell death and tissue damage (Ramesh and Saralakumari, 2012 ▶). In our study, administration of herbal combination and metformin showed marked improvement in MDA level, total thiol content and CAT activity compared to diabetic animals. The SOD activity was increased significantly in diabetic rats treated with herbal combination of dose 3 compared to diabetic rats. Impairment of antioxidant defense systems usually occurs in diabetes (Kumar et al., 2006 ▶). Pathogenesis of liver injury is closely associated with the oxidative stress (Wei et al., 2014 ▶). Excessive ROS production damages cellular macromolecules and structures leading to hepatic injury (Ullah et al., 2015 ▶). The antioxidant potential of *C. mukul* was well documented. Ramesh et al. reported a significant increase in SOD and CAT activity following treatment with *C. mukul* which resulted in rise of free radical scavenging activity (Ramesh and Saralakumari, 2012 ▶). Hyperglycemia is well known to increase ROS generation by glucose auto-oxidation and subsequent increment of lipid peroxidation (King and Loeken, 2004 ▶). *T. chebula* exhibited antioxidant capacity (Cheng, Lin et al., 2003 ▶) and a very high *in vitro *radical scavenging activity (Sasidharan et al., 2012 ▶). Protective effect of *C. myrrha* against liver oxidative damage caused by lead acetate was investigated in mice; it was argued that its polyphenolic compounds may induce protective effects against ROS production (El‐Ashmawy et al., 2006 ▶). In this investigation, the antioxidant properties of herbal combination against markers of oxidative stress was reflected by lower levels of MDA, higher total thiol content and increased activities of antioxidant enzymes SOD and CAT, in diabetic rats treated with the herbal combination.

In conclusion, the results of our study demonstrate that a combination of *C. mukul*,* C. myrrha* and *T. chebula* with antihyperglycemic, and lipid lowering and beneficial effect on scavenging free radicals could be considered for the development of novel drugs with high potential for reducing type II diabetes complications and management of its metabolic disturbance.
